# Looking Forward

**Published:** 1889-12

**Authors:** S. H. Preston


HALL'S
JOURNAL OF HEALTH

TRUTH DEMANDS NO SACRIFICE: ERROR CAN MAKE NONE.

VOL. 36. DECEMBER, 1889. No. 12

### LOOKING FORWARD.

BY S. H. PRESTON.

No. 4.

Dr. Babbitt believes that the evils of over-population are to be averted by co-operative colonies. Let us see.

A colony was established by the British government. It was in charge of competent officials, chosen with care by the crown. It was wholly composed of picked people. Transportation was furnished free. So were food, clothing, and all the necessaries of life. All were obliged to be frugal, industrious, regular in their habits, circumspect in their conduct and exactly equitable in all of their relations with each other and the government. Among them were no idlers, drones or drunkards. All promptly performed their allotted labor. Scrupulous justice, strict system, perfect harmony prevailed. A community could not be conducted on a better basis, or its business affairs be abler administered. And yet Botany Bay has generally been regarded as a good place to keep away from.

But Dr. B. refers to another bay, called Topolobampo, far off on the Mexican section of the Pacific coast. A colony is also there—a community composed of "integral co-operationists." "Pacific City" is there This "Paradise of the Pacific," as termed by its New York founders, who still live in New York, is thus described in circulars and press articles published by them last year :

"It is laid out in lots of 25x100 feet, contained in blocks 600 feet long by 300 feet wide. Avenues 200 feet in width ; streets 100 feet and walks of 50 feet, intersect one another at right angles ; while running diagonally, at an angle of 45 degrees in each direction, across the city plot, are wider avenues, called 'pradas,' to still further facilitate trade and travel. As thus arranged it is a model combination of urban and suburban beauty. Its wide streets and walks are lined by trees and bordered by broad belts of grass and flowers, while the whole is bounded by diversified and fertile farms, the common property of the citizens."

It is thus that the metropolis of this "model co-operative commonwealth," as mentioned in the printed matter of its non-resident promoters, appears on the map and to the mind's eye. The New York founders of the "Pacific City" are too busy embellishing maps, drawing diagrams of avenues and streets, putting "pradas" and parks in the proper places, and bordering them by "broad belts of grass and flowers," to become citizens of it themselves. I think Dr. Babbitt has no intention of removing there immediately. It may be an invasive but a valuable idea to him, that the attractions of this co-operative El Dorado appear to better advantage on a chart and to the mind's eye of the absent enthusiast than it does to the actual vision of the astonished visitant.

The tourist who turns up at Topolobampo, the site of "Pacific City," finds it tenanted by a few half-famished folks, rusticating in ragged tents. Their co-operative ability is devoted to keeping their souls in their bodies. They made their second anniversary the most memorable in their communal history by banquetting upon real meat. With all the varied sea-food of fifty-four miles of Pacific coast right before them, there appears to recur to these Topolobampobaynians an annual appetite for the flesh-pots of Egypt, or the tenderloin steaks of the United States. As seen from a rent tent on a vacant stomach, "Pacific City" seems more like a busted Gypsey camp than that "model of urban and suburban beauty," as pictured in the New York circulars and seen through the rose colored spectacles of the sanguine visionary.

Not a few foolish folks have found their way to this chart-created city and lived to get away. They do not expect to return 'till there are fewer "wide avenues, called pradas," and more markets. The oldest residents are those who have been unable to get away. Some supplies sent in from San Franciscans and charitable Mexicans have helped considerably in sustaining the connection between their souls and bodies.

The New York projectors pretend they have tried to prevent a rush of population to Pacific Paradise till they could build their railroads and boom up business a little better. To be sure they have already run one great trunk line through to Galveston that connects the Pacific with the Atlantic. That is, they have run it through as they have the flower-bordered streets of the city—on the map. They now only need the consent and co-operation of the Mexican government, an estimable number of millions of dollars and an indefinite term of years to put it in practical operation. It is believed that this railroad will be of great benefit to New Yorkers going to Topolobampo. It will also facilitate their speedy return.

But the most surprising thing in the prospectus of the Paradise projectors is this:—"The colony is incorporated under the laws of Colorado." A community in Mexico incorporated under the statutes of one of our States! This may be so the Mexicans could consider them as foreign foes in their own country in case of a rupture between their government and ours. Or perhaps they would not impress them into their army and confiscate their ragged tents out of respect for the laws of Colorado. In establishing a community in a foreign country it is always best to incorporate under the home laws of the corporators. This serves to show the sort of folks who are founding Topolobampo. Again they say:—

"Two hundred miles north are the great anthracite coal fields of Sonora, while one hundred and fifty miles to the east, stretches the vast lumber tract of Mexico, through which will pass the projected railway, thus bringing it to the door of industrial enterprise."

It must be, indeed, an intoxicating thought to these houseless, hungry tent-dwellers, that away one hundred and fifty miles to the east is a tract of timber they have no right to touch, and Sonora coal mines hundreds of miles to the north, and remind them of their own doorless dwellings rather than the "door of industrial enterprise." Verily Dr. Babbitt is right. Establishing Topolobampos would discourage population.

Now these Paradise planners are probably reformatory and philanthropic in their purposes. The trouble is their theory for turning Earth into an Eden cannot be carried into practical effect. Through all time there have been dreamers and community folks fidgeting the flesh off their bones in the vain endeavor to reconstruct society, and railroad the race right through to the millennium. Co-operation upon some plan has been their panacea for every social ill. Every conceivable phase of community life has been thoroughly tried and found a failure, except under phenomenally favoring circumstances and upon too small a scale to be of any service to society. As joint stock concerns under judicious business management, and with a profitable market for their manufactures, some few have prospered in a financial point of view, as the Shakers and Oneida community. But these owe their success to their peculiar situations and advantageous avenues of trade rather than to their co-operative relations. But the Shakers are dying out, and the "complex" family founded by Noyes is now no more, save as a state incorporated company of married members under another administration. Brook Farm, founded by folks famed for brains, business ability—men and women of culture, influence and means, had to be abandoned.

A community at home, necessarily small, is soon absorbed by superior surrounding elements. A colony cut off from the competitive world cannot last for lack of a trade outlet. Either intercourse or isolation has a fatal effect on communal life.

The central idea of the colonizationists is all right. It is a splendid scheme to unify and regenerate the race. But the race is not ripe for it. Human nature will not brook a social bit, though gilded with gold and leading to the fairest fields of earthly felicity. The wheels of progress are too wide and ponderous for any co-operative rut.

The Doctor refers to the "happifying and beautifying effect" of the Familistere of France, which after twenty-seven years of extraordinary effort has succeeded in providing for 2,000 people. He says this is "simply marvelous." Would we consider it "marvelous" for a missionary society to make only 2,000 converts in twenty-seven years, or an average of seventy-four per annum? At that rate how long will it take to Familistere the world, population doubling every twenty-five years according to Darwin? Should "Pacific City" be blessed with the same "simply marvelous" success, and its population increase at the rate of 2,000 every twenty seven years, at the end of 135 years it would contain 10,000 co-operative souls. Colonize the entire earth at that rate, and on the Topolobampo plan, and I agree with Dr. B. that sometime in the endless future it would prove an effectual remedy for over population.

It is a grand idea to gather up the destitute, depraved and distressed, and make them useful members of co-operative communities. But the experiment will always prove an expensive one. It is a philanthropic project to pick up poor and unemployed people here in New York and ship them away to "Pacific City" to work their share of some of the

"diversified and fertile farms, the common property of the citizens," as set forth in the aforesaid circular, and where they could soon sit down under their own vines and fig trees. But the cash cost. Figure what would be required to buy the land, farming implements, stock, seed, etc., beside to pay for putting up dwellings and passage tickets to Topolobampo. And after all this is paid for and the Bowery bum is put down at "Pacific City," he cannot go right out and sit down under his vine and fig tree. He must plow and plant and sow, and wait in his tattered tent for the crops to grow and ripen and be harvested. And then what will he do with them after reserving enough for his own needs? He must then borrow a few millions of dollars and join Jay Gould in running a railroad through to some available market. Much cheaper to put him in a mansion here on Madison Avenue and a fortune for him in the bank. It is cheaper to go to the nearest bakery and buy bread for a beggar than to buy a farm for him in a foreign country, pay his passage there and provide for him till he can raise his own wheat. And there is always a chance that after a man has been incorporated into a co-operative community that he wont co operate worth a cent. It has always been found in families that the smaller they are the better they co-operate.

As far as furnishing food, it is economy to send food to the folks who want it instead of sending the folks for the food. There are just so many mouths in the world at one time and just so much food for them. And were the entire pauper population of Europe transferred to Texas, the transaction would not give mankind one mouthful of bread the more, or reduce the demand for that bread by one mouth the less. The best way to prevent pauperism is to prevent such families as the Jukes from propagating paupers. More are bred here in Baxter street in one year than can ever be colonized at Topolobampo.

Until human nature is remolded and the established state of society remodeled, co-operative efforts will prove but feeble and futile ferments. Ages ago mankind outgrew the old gregarious condition of the original, barbarous races, and the deft fingers of human destiny are busy in these modern days unknitting the knots that have tied up individuality into septs and tribes, clans and communities. The machinery of a community may be all right, and the people to be put through it may be all right; but the productions of Phidias and Praxiteles were chiseled by hand, and the best types of humanity are not cast in molds like the fonts in the printer's case.

